# Prospective Newborn Screening for Sickle Cell Disease and Other Inherited Blood Disorders in Central Malawi

**DOI:** 10.3389/ijph.2021.629338

**Published:** 2021-06-29

**Authors:** Gerald Tegha, Hillary M. Topazian, Portia Kamthunzi, Thad Howard, Zondwayo Tembo, Tisungane Mvalo, Nelecy Chome, Wiza Kumwenda, Tawonga Mkochi, Arielle Hernandez, Kenneth I. Ataga, Irving F. Hoffman, Russell E. Ware

**Affiliations:** ^1^ University of North Carolina Project-Malawi, Lilongwe, Malawi; ^2^ Department of Epidemiology, University of North Carolina, Chapel Hill, NC, United States; ^3^ Institute for Global Health and Infectious Diseases, University of North Carolina, Chapel Hill, NC, United States; ^4^ Division of Hematology, Cincinnati Children’s Hospital Medical Center, Cincinnati, OH, United States; ^5^ Department of Pediatrics, University of North Carolina, Chapel Hill, NC, United States; ^6^ Center for Sickle Cell Disease, The University of Tennessee Health Science Center, Memphis, TN, United States; ^7^ Department of Pediatrics, University of Cincinnati College of Medicine, Cincinnati, OH, United States; ^8^ Global Health Center, Cincinnati Children’s Hospital Medical Center, Cincinnati, OH, United States

**Keywords:** newborn screening, sickle cell disease, sickle cell trait, alpha thalassemia, G6PD deficiency, Malawi

## Abstract

**Objectives:** Newborn screening in the United States and Europe allows early identification of congenital disorders but does not yet exist in most low-resource settings, especially in sub-Saharan Africa. Newborn screening can identify multiple inherited hematological disorders, but feasibility and effectiveness for Africa are not fully determined.

**Methods:** Surplus dried blood spot collected in Central Malawi through the HIV Early Infant Diagnosis surveillance program were repurposed and tested by isoelectric focusing for sickle cell disease and trait. Additional genetic testing identified G6PD deficiency and alpha thalassemia.

**Results:** Testing of 10,529 cards revealed an overall sickle cell trait prevalence of 7.0% (range 3.9–9.7% by district); 10 of 14 infants identified with sickle cell disease (prevalence 0.1%) were located and received care at a specialized clinic. Subsequent testing of 1,329 randomly selected cards identified alpha thalassemia trait in 45.7% of samples, and G6PD deficiency in 20.4% of males and 3.4% of females, with 29.0% of females as heterozygous carriers.

**Conclusion:** Inherited hematological disorders are common in Central Malawi; early identification through newborn screening can improve clinical outcomes and should be supported throughout Africa.

## Introduction

In high-income countries, newborn screening occurs for over 50 congenital health conditions, supported by government funding, public education, and trained health care workers [1]. However, in sub-Saharan Africa, newborn screening programs are limited in scope and may only screen for a single disease within restricted populations who have access to health care [2]. For conditions such as sickle cell disease (SCD), the global disparity in newborn screening neglects populations that are most at risk; 85% of SCD cases occur in Africa [3] where early-life mortality estimates range between 50 and 90%, especially in areas where there is limited clinical awareness of signs and symptoms, and where SCD diagnosis and treatment are scant or nonexistent [4]. In some developed countries, newborn screening for SCD is included in formal recommendations and national guidelines [5,6]; these national newborn screening programs have been developed through pilot programs which have compiled enough evidence to show that newborn screening is necessary for better health outcomes [7]. In Africa, standardized guidelines are not available, but an effort has been made in recent years to report the results of ongoing programs and to increase international collaboration [2,8], contributing to heightened awareness of SCD screening among decision makers in Ministries of Health.

In Malawi, the national prevalence of SCD was recently estimated to be 0.1% and the prevalence of sickle cell trait (SCT) to be 9.1% [9] with estimates varying by geographic area. Children in Malawi with SCD likely constitute a large proportion of deaths attributed to other leading causes of mortality such as malaria, anemia, acute respiratory infection, prematurity, bacteremia, and HIV/AIDS. The true burden of SCD is not known due to a lack of awareness and diagnostic testing, and small sample sizes limit the accuracy and generalizability of current and historical estimates. Since deaths due to SCD mostly occur in children under five years old, efforts to save lives must include early diagnosis and treatment. Many affected children are only diagnosed after they develop severe symptoms or may never reach a hospital setting before death; presumably, many die without ever having the proper diagnosis established.

In addition to SCD and SCT, α-thalassemia and glucose-6-phosphate dehydrogenase (G6PD) deficiency are inherited blood disorders that are common among Malawian children; a recent country-wide survey revealed that over 40% of children under five years have deletion of one or two α-globin genes (α-thalassemia trait) and 20% of males have G6PD deficiency [9]. Inherited hemoglobinopathies are associated with early death, as well as acute and chronic health conditions and loss of productivity [10]. However, no research has assessed the practicality of prospective newborn screening for SCD and other inherited blood disorders in Malawi, or demonstrated the feasibility of finding affected babies with subsequent linkage to clinical care [9,11,12].

Malawi has a well-established regionalized HIV Early Infant Diagnosis (EID) program that conducts DNA and/or RNA PCR analysis on dried blood spots (DBS) collected within 6 weeks of birth from HIV-exposed infants born to HIV positive mothers [13]. The objective of this study was to repurpose existing DBS from EID programs to conduct the first ever regional SCD surveillance study in Malawi, to estimate the prevalence of SCD and SCT across the Central region. We explore associations between SCT, SCD, and malaria, and further identify G6PD deficiency and α-thalassemia trait among our study population using genetic testing methods. Determining the prevalence and distribution of inherited blood disorders represents the first step toward designing and targeting interventions to screen, diagnose, and manage these diseases in at-risk populations.

## Methods

### Study Design and Population

The Central region of Malawi is home to 7.5 million people, representing 43% of the country’s total population. As part of the Malawi EID program, HIV-exposed infants are tested for HIV at their first postnatal visit, usually at approximately 6 weeks of age, as part of the prevention of mother-to-child transmission program. The current study used existing, surplus DBS that were collected from children <24 months of age and tested for HIV at the Kamuzu Central Hospital (KCH), Partners in Hope, and Mzimba District Hospital Molecular Laboratories between May 2018 and December 2018. The study was approved by the National Health Sciences Research Committee at the Malawi Ministry of Health, the University of North Carolina at Chapel Hill (UNC) Institutional Review Board, and the Cincinnati Children’s Hospital Institutional Review Board.

Most SCD testing and care in the Central region takes place at KCH, a 1,000-bed, public tertiary care hospital operated by the Malawi Ministry of Health that serves a population of nearly 4 million people. A SCD clinic is held at KCH one day each week where approximately 500 children with SCD are enrolled in treatment and follow-up [14].

### Sample Collection

Clinicians, nurses, and laboratory managers from nine district hospitals, one tertiary hospital, and one private hospital in the Central Region were trained with instructions and educational materials to inform EID families that their children would be tested for SCD alongside HIV and notified and referred to clinical services if results were SCD positive. Parents of children with SCT were not notified, as those with a single abnormal HbS gene are largely asymptomatic carriers and are relatively protected from malaria infection, compared to normal children [15]. Five laboratory technicians completed on-site training for isoelectric focusing (IEF) operation and interpretation throughout the course of the study, and 33 selected clinicians and nurses from 11 health facilities in the catchment area were trained in SCD management for positive cases.

To test for HIV in the EID program, several drops of whole blood were collected from the infant’s heel onto Whatman 903 DBS protein saver filter paper. DBS cards were labeled with the date of collection and a unique identifying number, and demographic information was collected into an electronic data system. The specimens were air dried for a minimum of 4 h at room temperature. DBS from local health facilities were sent to dispatch hubs, where they were collated and forwarded to one of the three molecular laboratories participating in the study. Once DBS were tested for HIV, the cards were then sent to the UNC Project laboratory and repurposed for SCD testing. Each infant was linked to his/her accompanying DBS sample using unique identifying numbers and the demographic data from the EID electronic laboratory record.

### Laboratory Procedures

A small (∼3 mm) punch was removed from each DBS for the purpose of hemoglobin analysis using IEF gel analysis (PerkinElmer, Inc.) to test for HbS and other variant hemoglobins. Each punch was placed into a 96-well plate, followed by elution of hemoglobin using Resolve systems Hb Elution solution and electrophoresis using pre-cast agarose gels. IEF equipment and supplies were provided by PerkinElmer, Inc., and training was conducted by experienced technicians from Cincinnati Children’s Hospital. IEF gel bands were read and interpreted by trained laboratory technicians and reviewed by supervisory personnel. Children with SCD were traced by obtaining a parent’s contact information through registers at the testing health facilities and children were subsequently referred for management and care at KCH. Further testing of family members was not conducted.

After hemoglobin analysis, a subset of samples was sent to Cincinnati Children’s Hospital for testing of G6PD deficiency and α-thalassemia trait. The subset included all SCD cases and a random selection of up to 50 SCT and 50 normal samples within each stratum of sex and district. Samples were shipped on dry ice and kept frozen at −80°C until use. Genomic DNA analysis was performed as previously described [16,17] with accurate PCR-based techniques used to identify the presence of one-gene or two-gene deletional α-thalassemia (3.7 kb rightward deletion), as well as the presence of the G6PD A^−^ variant that is common in African settings.

### Statistical Analysis

Prevalence measures were calculated by dividing SCD, SCT, α-thalassemia trait, and G6PD deficiency case numbers over the total sample size within each district. Significance tests were computed using Pearson’s chi-square tests at α = 0.05. Historical malaria prevalence estimates, (*Plasmodium falciparum* in children ages 1–10 years) were taken from modeled population-based, community-level data collected from 1970 to 2001 at 73 survey locations across Malawi [18].

All analyses were conducted in SAS 9.4 (SAS Institute Inc., Cary, NC, United States), R 3.5.1 (R Foundation for Statistical Computing, Vienna, Austria), and QGIS 3.4.0 (Open Source Geospatial Foundation Project).

## Results

A total of 10,529 DBS samples were analyzed and linked to clinical and demographic data. Samples were evenly distributed by sex with 5,177 (49.2%) female and 5,082 (48.3%) male participants, and 270 (2.5%) with unknown sex. Among the 10,529 DBS, 14 (0.1%) were classified as SCD, 741 (7.0%) as SCT, and 9,774 (92.8%) as normal (Table 1). There were no significant differences in the prevalence of either SCD or SCT by sex. The vast majority (9,615/10,529, 91.3%) of samples were HIV negative and all 14 SCD samples were HIV negative. Of the 1,329 samples tested for α-thalassemia, 506 (38.1%) had deletion of one α-globin gene, -α/αα, and 102 (7.7%) had deletion of two α-globin genes, -α/-α. About one-fifth of the males (134/657, 20.4%) were G6PD deficient, as were a smaller number (23/683, 3.4%) of females. Almost one-third of females (198/683, 29.0%) were heterozygous carriers of G6PD deficiency.

**TABLE 1:** Characteristics of Malawi Sickle Surveillance Study participants in the Central region of Malawi, 2018, stratified by sickle cell, α-thalassemia, and G6PD status. Clinical data were linked with hemoglobin electrophoresis and PCR results from dried blood spots obtained from infants ≤6 weeks of age from Malawi’s HIV Early Infant Diagnosis program. Bold values are absolute number of infants and percentage in brackets.

	Sickle cell				α-thalassemia				G6PD males			G6PD females			
	SCD *n* (%)	SCT n (%)	Normal *n* (%)	Total n (%)	2 copies -α/-α *n* (%)	3 copies -α/αα *n* (%)	4 copies αα/αα *n* (%)	Total *n* (%)	Deficient: A *n* (%)	Normal: G *n* (%)	Total *n* (%)	Deficient: A *n* (%)	Carriers: AG *n* (%)	Normal: G *n* (%)	Total *n* (%)
Sample size	14 (0.1)	741 (7.0)	9,774 (92.8)	**10,529**	102 (7.7)	506 (38.1)	721 (54.3)	**1,329**	134 (20.4)	523 (79.6)	**657**	23 (3.4)	198 (29.0)	462 (67.6)	**683**
Sex															
Female	4 (0.1)	376 (7.3)	4,797 (92.7)	**5,177 (49.2)**	55 (8.1)	264 (38.9)	359 (52.9)	**678 (51.0)**	—	—	—	—	—	—	—
Male	10 (0.2)	344 (6.8)	4,728 (93.0)	**5,082 (48.3)**	47 (7.2)	242 (37.2)	362 (55.6)	**651 (49.0)**	—	—	—	—	—	—	—
Not provided	0 (0.0)	21 (7.8)	249 (92.2)	**270 (2.6)**	—	—	—	—	—	—	—	—	—	—	—
HIV status															
Positive	0 (0.0)	43 (7.5)	532 (92.5)	**575 (5.5)**	5 (8.2)	22 (36.1)	34 (55.7)	**61 (4.6)**	8 (32.0)	17 (68.0)	**25 (3.8)**	2 (5.4)	10 (27.0)	25 (67.6)	**37 (5.4)**
Negative	14 (0.1)	670 (7.0)	8,931 (92.9)	**9,615 (91.3)**	95 (7.8)	463 (38.0)	662 (54.3)	**1,220 (91.8)**	121 (19.8)	489 (80.2)	**610 (92.8)**	20 (3.2)	180 (29.0)	420 (67.7)	**620 (90.8)**
Unknown^a^	0 (0.0)	28 (16.9)	311 (91.7)	**339 (0.0)**	2 (4.2)	21 (43.8)	25 (52.1)	**48 (3.6)**	5 (22.7)	17 (77.3)	**22 (3.3)**	1 (3.8)	8 (30.8)	17 (65.4)	**26 (3.8)**

a Indeterminate, discordant, new sample to be collected, blank.

**FIGURE 1:**
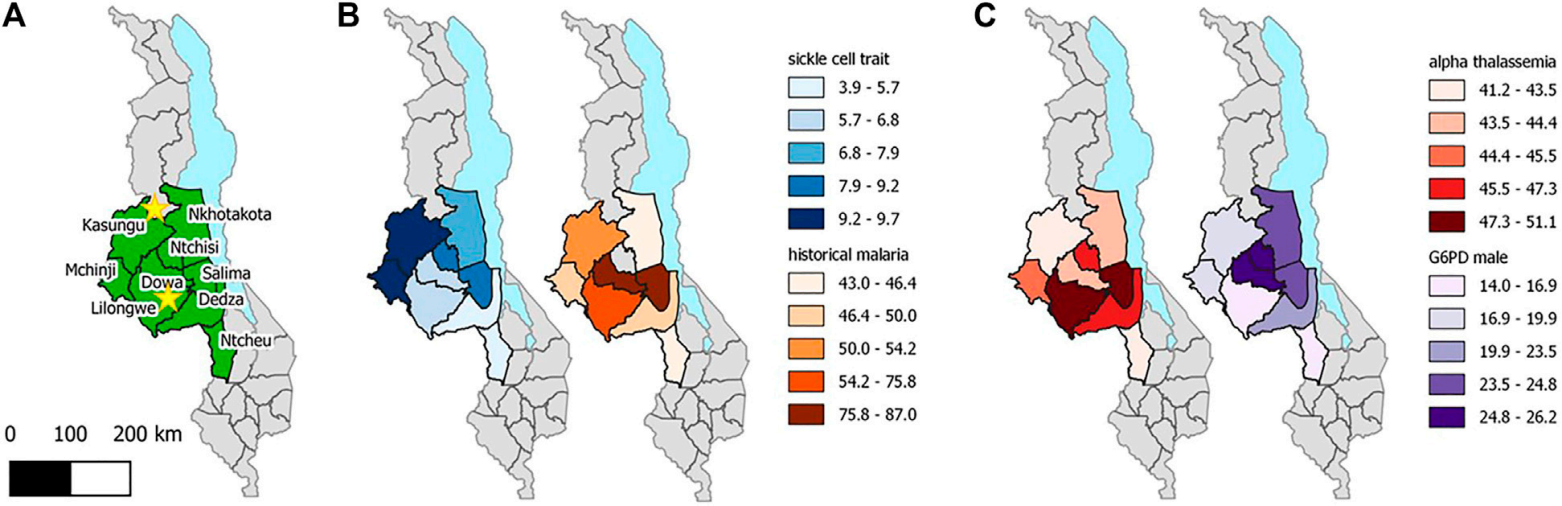
Malawi Sickle Surveillance Study prevalence estimates for sickle cell trait, historical malaria, α-thalassemia trait (-α/-α or -α/αα), G6PD deficiency in males, and G6PD deficiency/carrier status in females by district in the Central region of Malawi, 2018. Historical malaria prevalence estimates, (*Plasmodium falciparum* in children ages 1–10) are taken from data from 1970 to 2001 at 73 survey locations across Malawi [18]. Stars represent the location of Early Infant Diagnosis laboratory collaborators (Partners in Hope and Kamuzu Central Hospital in Lilongwe and Mzimba District Hospital in the North).

SCT prevalence was highest in the Northwest and lowest in the Southern part of Central region, ranging from 3.9% in Dedza to 9.7% in Kasungu (Figure 1). The highest SCT prevalence districts did not correspond closely with districts reporting the highest malaria prevalence in children, using historical data. However, malaria data were only available within the last few decades, limiting the extent of inference to selective genetic pressures. Of the 14 SCD cases, 12 were from Lilongwe (0.3% of Lilongwe samples), one was from Mchinji, and one from Ntcheu (Table 2). The prevalence of α-thalassemia trait (-α/-α or -α/αα) ranged from 41.2% (56/136) in Ntcheu to 51.1% (94/184) in Salima. G6PD deficiency prevalence among males ranged from 14.0% (15/107) in Lilongwe to 26.2% (16/61) in Dowa. G6PD deficiency or G6PD heterozygous carrier status was lowest in Ntchisi (13/57; 22.8%) and highest in Salima (42/92, 45.7%).

**TABLE 2:** Prevalence of inherited hematological disorders in the Malawi Sickle Surveillance Study, stratified by district in the Central region of Malawi, 2018. Historical malaria prevalence estimates, (*Plasmodium falciparum* in children ages 1–10) are taken from data from 1970 to 2001 at 73 survey locations across Malawi [18]. Bold values are absolute number of infants and percentage in brackets.

		Malaria	Sickle cell				α-thalassemia^a^				G6PD males^b^			G6PD females^c^			
	District	Prevalence 1970–2001	SCD *n* (%)	SCT *n* (%)	Normal *n* (%)	Total *n* (%)	2 copies -α/-α *n* (%)	3 copies -α/αα *n* (%)	4 copies αα/αα *n* (%)	Total *n* (%)	Deficient: A *n* (%)	Normal: G *n* (%)	Total *n* (%)	Deficient: A *n* (%)	Carriers: AG *n* (%)	Normal: G *n* (%)	Total *n* (%)
1	Dedza	0.50	0 (0.0)	28 (3.9)	684 (96.1)	**712 (6.8)**	12 (9.6)	45 (36.0)	68 (54.4)	**125 (9.4)**	12 (21.4)	44 (78.6)	**56 (8.5)**	1 (1.4)	17 (24.6)	51 (73.9)	**69 (10.1)**
2	Dowa	0.87	0 (0.0)	24 (6.6)	341 (93.4)	**365 (3.5)**	6 (5.0)	46 (38.7)	67 (56.3)	**119 (9.0)**	16 (26.2)	45 (73.8)	**61 (9.3)**	2 (3.4)	13 (22.0)	44 (74.6)	**59 (8.6)**
3	Kasungu	0.53	0 (0.0)	70 (9.7)	650 (90.3)	**720 (6.8)**	14 (9.6)	49 (33.6)	83 (56.8)	**146 (11.0)**	12 (17.6)	56 (82.4)	**68 (10.4)**	4 (5.1)	17 (21.5)	58 (73.4)	**79 (11.6)**
4	Lilongwe	0.59	12 (0.3)	318 (6.7)	4,427 (93.1)	**4,757 (45.2)**	18 (8.6)	82 (39.2)	109 (52.2)	**209 (15.7)**	15 (14.0)	92 (86.0)	**107 (16.3)**	3 (2.9)	29 (28.2)	71 (68.9)	**103 (15.1)**
5	Mchinji	0.50	1 (0.1)	110 (9.6)	1,039 (90.3)	**1,150 (10.9)**	14 (7.6)	69 (37.5)	101 (54.9)	**184 (13.8)**	17 (19.5)	70 (80.5)	**87 (13.2)**	3 (3.1)	30 (30.6)	65 (66.3)	**98 (14.3)**
6	Nkhotakota	0.44	0 (0.0)	29 (7.3)	366 (92.7)	**395 (3.8)**	6 (5.3)	44 (38.9)	63 (55.8)	**113 (8.5)**	14 (24.6)	43 (75.4)	**57 (8.7)**	1 (1.8)	20 (35.1)	36 (63.2)	**57 (8.3)**
7	Ntcheu	0.43	1 (0.1)	42 (4.4)	906 (95.5)	**949 (9.0)**	9 (6.6)	47 (34.6)	80 (58.8)	**136 (10.2)**	11 (15.9)	58 (84.1)	**69 (10.5)**	2 (2.9)	24 (34.8)	43 (62.3)	**69 (10.1)**
8	Ntchisi	*NA*	0 (0.0)	14 (8.9)	143 (91.1)	**157 (1.5)**	11 (9.7)	42 (37.2)	60 (53.1)	**113 (8.5)**	14 (25.0)	42 (75.0)	**56 (8.5)**	1 (1.8)	12 (21.1)	44 (77.2)	**57 (8.3)**
9	Salima	0.87	0 (0.0)	106 (8.0)	1,218 (92.0)	**1,324 (12.6)**	12 (6.5)	82 (44.6)	90 (48.9)	**184 (13.8)**	23 (24.0)	73 (76.0)	**96 (14.6)**	6 (6.5)	36 (39.1)	50 (54.3)	**92 (13.5)**

a *n* tested = 1,329.

b *n* tested = 657.

c *n* tested = 683.

The presence of SCT appeared to be inversely associated with G6PD trait in males; 22.1% (97/439) of male infants without SCT had G6PD deficiency, while 16.7% (35/209) of SCT males were G6PD deficient, however, these estimates did not reach statistical significance (*p* = 0.1) (Table 3).

**TABLE 3:** Sickle cell disease, sickle cell trait, α-thalassemia deficiency, and G6PD deficiency and carrier status prevalence estimates among Malawi Sickle Surveillance study participants, 2018, stratified by genotype.

		α-thalassemia^a^				G6PD males^b^			G6PD females^c^			
		2 copies -α/-α *n* (%)	3 copies -α/αα *n* (%)	4 copies αα/αα *n* (%)	Total *n* (%)	Deficient: A *n* (%)	Normal: G *n* (%)	Total *n* (%)	Deficient: A *n* (%)	Carrier: AG *n* (%)	Normal: G *n* (%)	Total *n* (%)
Sickle cell	Disease	1 (7.7)	3 (23.1)	9 (69.2)	13 (1.0)	2 (22.2)	7 (77.8)	9 (1.4)	—	—	4 (100.0)	4 (0.6)
	Trait	32 (7.3)	167 (38.2)	238 (54.5)	437 (32.9)	35 (16.7)	174 (83.3)	209 (31.8)	9 (3.8)	68 (29.1)	157 (67.1)	234 (34.3)
	Normal	69 (7.8)	336 (38.2)	474 (53.9)	879 (66.1)	97 (22.1)	342 (77.9)	439 (66.8)	14 (3.1)	130 (29.2)	301 (67.6)	445 (65.2)
α-thalassemia	-α/-α	—	—	—	—	9 (19.1)	38 (80.9)	47 (7.2)	3 (5.5)	17 (30.9)	35 (63.6)	55 (8.1)
	-α /αα	—	—	—	—	45 (18.6)	197 (81.4)	242 (36.8)	10 (3.8)	75 (28.4)	179 (67.8)	264 (38.7)
	αα/αα	—	—	—	—	80 (22.1)	282 (77.9)	362 (55.1)	10 (2.8)	102 (28.4)	247 (68.8)	359 (52.6)
	No score	—	—	—	—	—	6 (100.0)	6 (0.9)	—	4 (80.0)	1 (20.0)	5 (0.7)

a *n* tested = 1,329.

b *n* tested = 657.

c *n* tested = 683.

Of the 14 cases of SCD, 10 babies were successfully traced and brought for management and care at the KCH SCD Clinic. This included verification of disease by repeat isoelectric hemoglobin electrophoresis for SCD, initiation of folic acid supplements, penicillin prophylaxis, pneumococcal vaccination, malaria prophylaxis with sulfadoxine-pyrimethamine, and the opportunity to receive disease-modifying treatment with hydroxyurea. The four remaining cases of SCD were unable to be traced; one relocated to Blantyre, and three could not be reached due to a missing register log at one of the clinics that contained the patients’ contact information.

## Discussion

Newborn screening for SCD and other hemoglobin disorders is now universal in the United States, with excellent results and outcomes [19]. Despite infrastructure limitations, small urban newborn screening programs for SCD have proven to be feasible in Ghana [20], Benin [21], Nigeria [22], and the Democratic Republic of Congo [23]. Comparable to resource-rich countries, early diagnosis followed by active management of affected children has also shown decreased mortality in Benin and Jamaica [24]. Pilot data from the Republic of Angola documented a high SCD incidence, with successful location and retrieval of affected babies allowing initiation of early life-saving interventions including penicillin prophylaxis, pneumococcal vaccines, insecticide-treated bed nets, and parental education [25]. Hydroxyurea has recently been shown to have efficacy for children with SCD living in sub-Saharan Africa [26,27], and should also become part of the clinical management of children identified with SCD through newborn screening programs.

We documented the prevalence of inherited hematological disorders in the Central region as follows: 0.1% SCD, 7.0% SCT, 45.7% α-thalassemia trait, 20.4% G6PD deficiency in males, and 29.0% G6PD female carriers. Within the region, SCT prevalence varied by district, ranging from to 3.9–9.7%, with the highest prevalence on the western border with Zambia and the southern border with Mozambique. Variation within the region is similar to that observed in Uganda, where the prevalence of SCT varied 3–5 fold across the country [28].

The prevalence of SCD among our study population, 0.1%, is within the lower bound of prior estimates found among infants and preschool children in Malawi, which range from 0.04 to 2.5% [9,11,12]. Our SCT prevalence of 7.0% is slightly lower than that the 9.1% SCT figure recently cited in a national secondary analysis of children under the age of five in Malawi, and prevalence of α-thalassemia and G6PD deficiency were similar [9]. The burden of SCD in Malawi has been underappreciated as a source of childhood morbidity and mortality, but is also lower than estimates found in the neighboring countries of Tanzania (0.6%) and Zambia (0.9%) [2,29]. However, prevalence measures in Tanzania are also known to vary geographically; preliminary data using EID samples from Northern Tanzania found a 20.8% prevalence of SCT [30], while lower estimates of SCT down to 9.6% have been found in areas in and around Dar es Salaam [31].

α-thalassemia trait in our study cohort also varied geographically, from 41.2% to 51.1% by district, confirming a high allele frequency of the 3.7 kb rightward deletion in Eastern Africa. A prior analysis of Malawian children tested in a smaller country-wide anemia survey [9] documented a 43% prevalence of α- thalassemia trait (33% -α/αα and 10% -α/-α), which is similar to our findings in the Central region. We noted substantial variation within the region with higher prevalence in the south, analogous to observations in Uganda where the prevalence of α-thalassemia trait increased toward the eastern part of the country [17].

Similarly, the prevalence of G6PD deficiency was high in the Central region of Malawi, with one-fifth of males affected and almost one-third of females (29.0% carriers, 3.4% affected). As with the other hematological disorders, the prevalence varied across the Central region, with district prevalence ranging from 14.0% to 26.2% among males. The prevalence of G6PD deficiency is higher than published values for Uganda, where 14% of males were affected overall, and an increasing prevalence toward the southern portion of the country [17].

Decades ago, Haldane posited his malaria hypothesis that maintaining a high frequency of genetic hematological disorders is the advantage gained by the affected individual for malaria survival [32]. Especially for SCT, varying geographical differences relates to the distribution of *P. falciparum* malaria; persons with SCT have been shown to be less likely to acquire malaria infection and even less likely to die of severe malaria [33–35]. However, the SCT prevalence within the Central region of Malawi did not correlate with historical patterns of malaria prevalence from 1970 to 2001 (Figure 1), perhaps because of advances in malaria diagnosis and treatment; strong genetic selection pressure has likely been superseded by migration patterns and malaria control measures over the past 20 years [36]. A smaller national survey in Malawi showed similar results; SCT prevalence by region did not align with malaria prevalence either by rapid diagnostic test or by self-report of recent infection [9].

Importantly, the prevalence of these three hematological conditions that confer protection against malaria were not well correlated across the Central region of Malawi, and the highest prevalence of each trait was found in different districts. This lack of correlated prevalence reflects the tendency for individual protective traits to negate the effects of another. Such negative epistasis has been reported between SCT and α-thalassemia in Kenya [37], and between SCT and G6PD deficiency in Mali [38], with no additive protection and even loss of protection against malaria infection. To our knowledge, this is the first report of all three protective traits plotted with the same geographical distribution, which supports the concept of negative epistasis for malaria in Central Malawi.

α-HIV EID programs, which exist in all 46 PEPFAR countries, should be considered an available and feasible mechanism for introducing newborn screening to determine SCD prevalence, while simultaneously acting as a physical infrastructure platform for treatment services. SCD is a lifelong chronic condition requiring consistent treatment with penicillin, hydroxyurea, and intermittent acute care for severe events such as blood transfusions, and using existing systems such as immunization and HIV early care programs can be a sustainable and essential way to retain young adults and children in care [2,39]. While screening is the first step in understanding the burden of disease, treatment is necessary to reduce morbidity and mortality from SCD. A recent study reporting on baseline clinical and laboratory characteristics of children confirmed with SCD at KCH in Central Malawi found that patients had substantial morbidity, with a large percent reporting histories of anemia (71.8%), jaundice (52.1%), joint pain (56.4%), and pain episodes (49.6%) [40]. A remarkable 74.4% had received at least one previous blood transfusion, which stresses the need for improving and standardizing approaches to transfusion therapy for children with SCD, especially in a setting with limited blood supply. Implementation of hydroxyurea has already been shown to be safe and effective, with clinical benefits for high-risk children with SCD in our setting [14]. Currently, KCH is the only clinic in the Central region that has specific SCD management, and transportation and distance are barriers to patient referral. Education for district clinicians on SCD treatment and management are key components of a scale-up of a national screening program, incorporating training to identify and test anemic hospitalized children to locate those at high-risk of underlying SCD.

A major strength of this study is the use of existing DBS and a centralized laboratory testing system, demonstrating a mechanism that could be scaled-up to create a national testing program. Additionally, this is the first regional SCD surveillance study in Malawi, collecting samples from all districts in the Central region, and showing variation in the prevalence of SCD, SCT, α-thalassemia trait, and G6PD deficiency over a broad geographical area. Distribution patterns of genetic factors of disease provide justification for improved laboratory capacity and for the expansion of both newborn screening programs and SCD diagnosis and treatment infrastructure. A broader scale-up of our methods could successfully connect infants to care, potentially leading to better health outcomes and lower mortality.

This study was limited by the small number of SCD cases. Testing of over 10,000 samples only yielded 14 SCD cases, however our sample size is much larger than previous surveillance studies showing the effectiveness of newborn screening programs elsewhere in sub-Saharan Africa [31,41]. Limitations to generalizability in a broader population could include selection bias stemming from our sampling of HIV-exposed infants through Malawi’s EID program. Mothers may be more likely to bring infants to be tested who are sicker, whether from HIV or SCD related symptoms. Despite this potential bias, EID programs are a good starting point for SCD screening, given the hypothesized increased morbidity of SCD in HIV infected infants [17]. Children with SCD were followed up, but parents of children with other inherited blood disorders were not contacted, as α-thalassemia trait does not require specific medical intervention, and primaquine, the main source of morbidity for G6PD deficient children, is not commonly used in Malawi.

A final consideration relates to the burden of disease and the likelihood of creating a feasible and cost-effective newborn screening programs in sub-Saharan Africa. The Central region of Malawi has an approximate crude birth rate of 32.5 births per 1,000 population, with an estimated 244,600 children born to mothers in the Central region during the 12 months prior to the 2018 census [42]. Using the disease rates detected in our study, we would expect approximately 325 children in the Central region to be born each year with SCD and 17,214 children to be born each year with SCT. The high prevalence of the HbS allele in sub-Saharan Africa, where overall under-5 mortality from other causes is improving, suggests that the relative contribution of SCD to under-5 mortality is likely to increase significantly and steadily over the coming years [43]. Affected babies with SCD, who would previously have died early in life from other causes, may now be surviving long enough to present for diagnosis and develop serious morbidity that requires care and treatment [44]. The results from our study indicate that developing a newborn screening program is feasible in Malawi, both to conduct disease surveillance, and to locate children with SCD and integrate them into care. Newborn screening is the first step to improving existing care and treatment programs to incorporate SCD management based on the geographic burden of disease, targeting highly affected districts for introduction of an integrated community-based diagnosis, care, and treatment program.

## Data Availability

The original contributions presented in the study are included in the article/supplementary material, further inquiries can be directed to the corresponding author.
